# Navigating fragmented and contingent support: A qualitative study of British Armed Forces families raising neurodivergent children

**DOI:** 10.1371/journal.pone.0358595

**Published:** 2026-09-21

**Authors:** Niko Kargas, Mirena Dimolareva, Stamatina Tsiora, Anum Farooq, Victoria L. Brelsford, Holly Stocks, Ella Doolan-Dransfield, Julian Free

**Affiliations:** 1 Department of Cognitive Sciences, United Arab Emirates University, Al Ain, United Arab Emirates; 2 School of Psychology, Sports Science and Wellbeing, University of Lincoln, Lincoln, United Kingdom; 3 Psychology and Language Sciences, University College London, London, United Kingdom; 4 Vice Chancellor’s Office, University of Lincoln, Lincoln, United Kingdom; Nagoya University: Nagoya Daigaku, JAPAN

## Abstract

British Armed Forces families raising neurodivergent children must coordinate health, education, welfare and informal support within a service context characterised by mobility and deployment. This study examined how families understood, accessed and sustained support, and how these processes shaped caregiving and family life. Twelve parents and caregivers of children aged 3–17 years with diagnosed or suspected neurodevelopmental conditions participated in online semi-structured interviews. Guided by critical realism, data were analysed using reflexive thematic analysis. The analysis showed that support was experienced less as a stable entitlement than as a contingent achievement. Relocation repeatedly interrupted clinical, diagnostic and educational pathways. Parents consequently became continuity brokers who carried records, rebuilt professional relationships and repeatedly legitimised their children's needs. Military welfare and informal networks could provide meaningful practical and emotional help, but access depended heavily on local knowledge, individual champions and geographic proximity. Advocacy generated occupational, financial and emotional costs that were often gendered. Deployment further redistributed care, disrupted routines and reverberated across caregiver wellbeing, child regulation and serving personnel's work. Parents also described meaning, pride and solidarity, while resisting uses of “resilience” that shifted responsibility from systems to families. These findings identify a policy-implementation gap: commitments to avoid service-related disadvantage do not consistently translate into portable, neurodiversity-informed support. Greater continuity across postings, transparent inter-agency handovers, knowledgeable welfare provision and deployment-sensitive respite are required.

## Introduction

Families in the UK Armed Forces experience distinctive stressors that differentiate them from civilian households, including frequent mobility, parental separations, and the persistent risk associated with military service [1–3]. For families raising neurodivergent children, these features of service life intersect with the need for stable routines, specialised educational provision, and timely access to diagnostic and therapeutic services, generating a dense “care ecology” that must be continuously renegotiated as postings and deployments change.

Neurodevelopmental conditions such as autism spectrum disorder (ASD) and attention-deficit/hyperactivity disorder (ADHD) are typically conceptualised as early-emerging, lifelong differences in social communication, behaviour, attention and information processing [4,5]. In England, population-based studies suggest that around 3% of children aged 10–14 have an autism diagnosis, with evidence of substantial under-diagnosis in primary care records [6], while broader estimates suggest that 10–20% of children may be neurodivergent when ADHD and related developmental conditions are included [5,7]. For ADHD, global prevalence in childhood is approximately 5% [8].

Terminology in this study is informed by the neurodiversity paradigm [9–12]. We use neurodiversity to describe variation across a population and neurodivergent to describe individuals whose cognitive or developmental characteristics diverge from dominant norms. This position does not require a choice between “difference” and “disability.” Participants’ children could experience valued forms of difference alongside substantial disability, co-occurring health needs and support requirements, particularly where environments and services were inaccessible. Clinical terms are retained when they were used in diagnostic pathways, service eligibility or participants’ own accounts. We therefore avoid treating neurodivergence as inherently deficient while recognising the material consequences of unmet needs and disabling contexts.

Beyond prevalence, research consistently documents the intensive demands placed on caregivers of autistic and otherwise neurodivergent children, including heightened stress, reduced leisure, and constrained employment and community participation [13–15]. These demands are amplified when children present with complex co‑occurring conditions, such as epilepsy or avoidant/restrictive food intake disorder (ARFID), which further increase care coordination and emotional labour [16,17].

### Formal and informal support for armed forces families

Formal support refers to structured services delivered by trained professionals, including healthcare, education, social care and military welfare. Such support can provide information, care coordination and problem-solving skills for parents [18,19]. However, Armed Forces families often face disruptions in access due to frequent relocations and deployments, which can interrupt established relationships with clinicians and educators and reset waiting lists [20,21]. For families of children with special educational needs and disabilities (SEND), specialist schools and services are not always located near military bases, which can be in remote areas. This contributes to inequities in provision and to what parents describe as a “postcode lottery” [22,23].

For this study, support was treated as more than the formal existence of a service or the presence of a social contact. Support was analytically understood as information, practical assistance, care coordination, advocacy, accommodation or emotional help that was accessible, usable, responsive to the child and family, and sufficiently continuous to reduce rather than transfer coordination, risk and problem-solving work onto caregivers. This functional definition allowed the analysis to distinguish nominal provision from support that families experienced as effective.

Three military-specific policy instruments provide important but distinct context. The Armed Forces Covenant expresses the principles that members of the Armed Forces community should not face disadvantage arising from service and that special provision may sometimes be justified [24–26]. During the study period, the associated legal duty required specified public bodies to give due regard to these principles when exercising relevant healthcare, education and housing functions; it did not create an automatic entitlement to a particular assessment, waiting-list position or school placement [26]. The Service Pupil Premium (SPP) is additional funding for eligible state-funded schools in England, intended principally to mitigate the effects of mobility and parental deployment rather than to replace statutory SEND provision [27]. The UK Armed Forces Families Strategy 2022–2032 is a broader strategic framework for improving family experience across service life [28]. These distinctions are important because the present study examines not simply whether frameworks existed, but whether their principles were translated into accessible and portable support in families’ everyday encounters with local systems.

Informal support, in contrast, comprises emotional, practical, and informational assistance from family, friends, neighbours, peers, and community networks [29–31]. Social support can buffer stress and promote wellbeing in families facing chronic adversity [32,33]. Within military settings, spouses and partners often rely heavily on informal networks, including other military families, to manage childcare, emotional strain, and the practical realities of deployment [2,34,35]. However, relocation can sever these informal ties, leaving families with minimal local support just as formal systems are disrupted.

### Neurodiversity and military family life

Military families raising neurodivergent children remain underrepresented in research. Existing studies suggest that these families face additional stressors beyond those experienced by either civilian neurodivergent families or non-SEND military households. Davis and Finke [36], for example, found that frequent relocations disrupted access to services for US military families raising autistic children, resulting in repeated diagnostic procedures, loss of specialist provision and advocacy fatigue. Research with Canadian and US samples similarly indicates that waiting lists for specialist services may exceed the duration of a posting and that service disruption can be associated with behavioural instability in children and psychological strain for caregivers [3,37,38].

In the UK, emerging work suggests that neurodivergent children in Armed Forces families and their caregivers face intertwined challenges of diagnostic delay, fragmented educational support and extended responsibility for care [21,39–41]. The Forces Additional Needs and Disability Forum (FANDF) reported that 48% of surveyed military families felt that their children's disabilities prevented them from living what they would consider a “normal” family life [42]. Research on special educational needs further highlights the emotional demands of navigating provision and parents’ continuing efforts to secure suitable schooling and support [43–45].

Taken together, existing research identifies high coordination demands in families raising neurodivergent children and recurrent disruption in mobile military populations, but provides limited UK evidence on how families judge whether support is usable, how continuity is maintained across local systems, and who absorbs the work when formal handovers fail. The present study addresses this process-level gap.

Critical realism provided the study's epistemological orientation: participants’ accounts were understood as situated interpretations of experiences occurring within material organisational, geographical and policy conditions [46]. Bronfenbrenner's ecological systems theory was used as a later interpretive lens to examine connections across the child and family, relationships with schools and clinicians, military units and welfare organisations, and wider policy and cultural contexts, including change over postings and deployments [47]. Family-systems concepts were applied more selectively to deployment, where absence, solo caregiving, child regulation and reintegration reverberated across family relationships [48–50]. Family stress and resilience scholarship informed interpretation of how demands, resources, coping and meaning-making interacted [51–55], while the analysis remained attentive to participants’ concern that resilience language can shift responsibility from institutions to families [56]. These frameworks sensitised interpretation; they were not used as a predetermined deductive codebook.

### The present study

The study aimed to examine how British Armed Forces families raising neurodivergent children understood, accessed and sustained formal and informal support within the intersecting contexts of mobility and deployment. It addressed three questions:

1. What did parents and caregivers regard as adequate and usable support for their neurodivergent children and families?
2. How did mobility, deployment and institutional arrangements shape access to and continuity of formal and informal support?
3. How did the work of securing and maintaining support shape parental wellbeing, resilience, occupational trajectories and family life?

## Methods

### Design and epistemology

This study employed a qualitative design using Braun and Clarke's reflexive thematic analysis to explore parents’ lived experiences of support within UK military life [57–59]. A critical-realist stance underpinned the analysis [46]. Participants’ accounts were treated as grounded in material conditions, including organisational policy, local service design and geographical mobility, while also being shaped by meanings and social discourses surrounding military service, caregiving and neurodivergence. The ecological, family-systems and stress-resilience frameworks described above informed later interpretation rather than the initial coding frame.

### Researcher positioning and reflexivity

The study was situated within a critical-realist framework [46], which recognises that participants’ accounts are shaped both by material conditions and by the meanings through which those conditions are interpreted and communicated. This position was well suited to a study seeking to understand how Armed Forces families experience, interpret and navigate support for neurodivergent children while attending to the institutional and structural conditions shaping those experiences. Interviews were conducted by three research team members who were themselves from British military families and had prior experience in qualitative research and neurodiversity-focused research. This background supported sensitive engagement with participants discussing caregiving demands, service-related mobility, educational disruption and the negotiation of formal and informal systems. It also created the possibility that familiarity with military culture or established assumptions about neurodivergence could shape questioning and interpretation.

Reflexivity was therefore integrated across data collection and analysis rather than treated as a separate quality check. Researchers maintained reflective notes and reflexive memos to examine how prior assumptions, disciplinary commitments, theoretical perspectives and responses to participants’ accounts might influence interpretation. Regular team discussions enabled emerging interpretations to be questioned, refined and considered in relation to the study's theoretical orientation and alternative explanations. Audit notes were maintained throughout analysis to document consequential analytic decisions, including the development, refinement and revision of codes, candidate patterns and thematic boundaries. These practices were used to make the interpretive process transparent and open to critical scrutiny. Researcher perspectives were not treated as distortions that could be eliminated, but as part of an interpretive process requiring ongoing reflexive engagement [58–60].

### Participants and recruitment

Twelve caregivers of neurodivergent children from UK Armed Forces families took part in semi-structured interviews. The sample included seven women and five men aged 30–48 years. Participants were serving personnel (n = 5), a veteran (n = 1), or spouses or partners of serving personnel (n = 6). Service contexts included the British Army and Royal Air Force, with participants also reporting Royal Logistic Corps and intelligence roles. Their children were aged 3–17 years and had formal diagnoses or were undergoing assessment for autism, ADHD, global developmental delay, avoidant/restrictive food intake disorder (ARFID), selective mutism, epilepsy and other co-occurring conditions, including dyspraxia and sensory processing differences. Aggregate sample characteristics are presented in Table 1.

**Table 1:** Aggregate participant characteristics.

Characteristic	Sample information
Participants	12 parents and caregivers
Gender	Seven women and five men
Age range	30-48 years
Relationship to service	Serving personnel (n = 5), veteran (n = 1), spouses/partners of serving personnel (n = 6)
Service contexts reported	British Army, Royal Air Force, Royal Logistic Corps and intelligence roles
Child age range	3-17 years
Child neurodevelopmental and co-occurring needs	Diagnosed or undergoing assessment for autism, ADHD, global developmental delay, ARFID, selective mutism, epilepsy and other co-occurring conditions

Rank, children's schooling arrangements, ethnicity, and socioeconomic position are not presented because combinations of these characteristics could increase the risk of deductive disclosure and compromise participant confidentiality.

Participants were initially recruited to complete an online survey through military networks, social media pages of Armed Forces support organisations, and participant referral, using a convenience- and volunteer-based recruitment approach. At the end of the survey, respondents were invited to indicate whether they were willing to be contacted for a follow-up interview by providing an email address. Twelve eligible respondents expressed interest in participating in an interview, provided consent to be contacted, met the study eligibility criteria, and completed an interview. As all eligible volunteers were invited and interviewed, no purposive sampling or participant selection procedures were applied. Recruitment for the online survey and subsequent interviews was conducted between 30 June 2024 and 15 July 2025, with interview data collection occurring within this period. Recruitment and data collection proceeded iteratively, as eligible survey respondents who indicated willingness to participate were subsequently contacted and scheduled for interview. The extended timeframe reflected the geographically dispersed and comparatively hard-to-reach nature of the Armed Forces population, as well as the time required for relevant military-related organisations to provide clearance for the circulation of recruitment materials through their networks. Contact information was collected and retained exclusively for the administration and scheduling of interviews and was stored in accordance with UK GDPR and institutional data governance requirements.

### Sample adequacy

The adequacy of the completed sample was evaluated using information power rather than a fixed numerical threshold [60]. The study addressed focused questions within a specific and comparatively hard-to-reach population. Interviews generated detailed accounts over approximately 60–90 minutes and the analysis was supported by an established literature and a specified contextual and epistemological frame. Across the 12 interviews, the dataset provided sufficient depth and variation to develop coherent patterned meanings concerning mobility, continuity work, contingent support and deployment while preserving differences between families. This judgement concerns adequacy for the bounded qualitative claims made here and does not imply statistical representativeness or exhaustive thematic saturation.

### Data collection

Semi-structured interviews were conducted online through Microsoft Teams to facilitate participation across geographically dispersed postings and to accommodate caregiving and service responsibilities. Interviews lasted approximately 60–90 minutes and followed a guide co-developed by the wider research team and informed by a systematic review and prior survey findings (Please see the S3 Interview Guide in S3 File for further details). Participants could choose between video-enabled or audio-only interviews. Field notes recorded notable pauses during interviews, but tone of voice and visible emotional expressions were not documented in the transcripts. Online interviewing increased accessibility for a dispersed and time-constrained population, although it provided less access than in-person fieldwork to embodied, spatial and interactional context. The analysis therefore focused on participants’ verbalised accounts and meaning-making and did not claim to provide a systematic analysis of gesture or body language.

Questions covered:

- Experiences of diagnosis and assessment (e.g., “Tell us about your experience of the diagnosis process. Any specific challenges?”).
- Parenting gratifications and challenges, including caregiving during deployment.
- Experiences within the military community, including perceptions of stigma or solidarity.
- Relocation experiences and impacts on education, healthcare and informal support.
- Formal support from the National Health Service (NHS), military welfare, education and charities.
- Informal support from family, friends and other military families.

The interview format was flexible, allowing participants to elaborate on emerging topics, particularly around high‑stakes issues such as school placements, tribunals, and care coordination.

### Transcription and data preparation

Interviews were audio-recorded with consent and transcribed verbatim. Identifying details, including specific locations, ranks, units and rare combinations of family circumstances, were removed or generalised during transcript preparation and reviewed again when quotations were selected. Initial transcripts were generated from Microsoft Teams recordings using available accessibility features. Transcripts were transcribed verbatim in NVivo, checked against the original recordings by NK and MD, corrected for substantive errors, and de-identified prior to analysis. NVivo was used to facilitate data storage, organisation, coding, and retrieval, while all interpretive analysis remained the responsibility of the research team.

### Ethical considerations

Ethical approval was obtained from the University of Lincoln Research Ethics Committee, UK (UOL2024_17640). Participants were fully informed about the study aims, procedures, confidentiality safeguards and their right to withdraw, and provided written informed consent before participation, including consent for audio-recording and the use of anonymised quotations. As interviews explored potentially distressing experiences related to caregiving strain, repeated relocation and institutional barriers, participants were offered information on relevant support organisations, including the Soldiers', Sailors' and Airmen's Families Association (SSAFA), NHS mental health services and Samaritans. All data were pseudonymised and stripped of identifiable markers, including specific locations, ranks and service-related identifiers. Because the study involved a small and potentially identifiable population, particular care was taken to minimise deductive-disclosure risk arising from combinations of service background, family circumstances, co-occurring conditions and location-specific details. A final disclosure review of all quotations was conducted before dissemination.

### Data analysis and analytic quality

Analysis followed Braun and Clarke's six-phase reflexive thematic analysis: familiarisation, generating initial codes, constructing themes, reviewing themes, defining and naming themes, and producing the report [57–59]. After repeated familiarisation, transcripts were coded systematically in NVivo. Coding was undertaken line-by-line, at the level of meaning-bearing units. Early coding remained primarily inductive and close to participants’ language, while later analytic work attended to more latent patterned meanings concerning continuity, legitimacy, responsibility and institutional design.

NK served as the lead analyst. The remaining members of the research team contributed to the analysis through critical review of candidate themes, discussion of alternative interpretations, and reflexive challenge of the developing analysis. Their involvement was intended to extend and interrogate interpretation rather than to establish coder consensus or calculate inter-rater agreement, consistent with the interpretive assumptions of reflexive thematic analysis [58,59].

Codes were compared across cases and clustered into candidate patterns. Candidate themes were developed around a central organising concept rather than a shared topic alone and were reviewed against coded extracts and the dataset as a whole. A de-identified example illustrating the route from participant extract to initial code, analytic interpretation, candidate pattern and final theme is provided in S1 Table. Each of the five final themes was checked against all 12 full transcripts. This complete-dataset review examined whether each theme represented a coherent pattern, remained distinguishable from neighbouring themes, addressed the research questions and adequately represented both recurrent and less common accounts.

Analytic quality was supported through repeated engagement with the complete dataset and through explicit consideration of the relationship between participants, quotations and interpretive claims. A participant-by-quotation audit of the Results extracts was completed to reduce unnecessary repetition, identify disproportionate reliance on particular speakers and ensure that each quotation performed a clear analytic function. Reporting was structured with reference to the Standards for Reporting Qualitative Research (SRQR) [61]. The completed 21-item SRQR checklist is provided as S2 File in the Supporting Information.

## Results

### Overview

The analysis organised families’ accounts around five interlocking themes. Across the dataset, support was experienced less as a stable resource than as a contingent achievement requiring ongoing work. Mobility made formal provision non-portable; parents became continuity brokers between systems; this work generated unequal emotional, occupational and financial costs; formal and informal support depended on relationships, knowledge and proximity; and deployment redistributed care and regulation across the family. Table 2 summarises the central organising concept of each theme. The themes are analytically distinct but mutually connected rather than independent categories.

**Table 2:** Themes and central organising concepts.

Theme	Central organising concept
1. Support made non-portable by mobility	Health, diagnostic, education and transport systems were organised around stable residence; moves disrupted queues, relationships, evidence and local provision.
2. Parents as continuity brokers	Parents carried information, rebuilt professional understanding, legitimised needs and connected services that did not reliably communicate.
3. The unequal costs of continuity work	Coordination and advocacy redistributed time, exhaustion, earnings, career opportunity and risk within families, with gendered patterns where supported by participants’ accounts.
4. Support as contingent relationships	Relatives, peers, welfare staff and commanders could be decisive, but help depended on individual knowledge, goodwill and geographic proximity rather than predictable design.
5. Deployment as redistribution of care and regulation	Absence and return reorganised routines, solo caregiving, child regulation, sleep, work and family equilibrium; informal respite sometimes buffered these effects.

Note: Quotations are reproduced from participant interviews and lightly shortened with ellipses where needed for readability. Participant identifiers are P1-P12.

### Themes and Interpretations

1 Support Made Non-portable by Mobility

Families consistently described relocation as a disruption not only of place, but of the administrative time, professional relationships and local evidence on which support depended. Health, diagnostic and educational systems were organised around stable residence, whereas service life required families to move between NHS trusts, local authorities and schools. Provision could therefore exist in principle while becoming unusable or inaccessible after a posting.

1.1 Health and Diagnostic Pathways That Restarted

Transfers between NHS trusts and local authorities frequently reset waiting lists, referrals and assessment processes. One parent summarised the experience as follows:

*“We had to start from square one... the Armed Forces Covenant didn’t apply to us.” (P2)*

This statement is presented as P2's experience of practical non-application rather than a legal conclusion. The Covenant's due-regard duty did not guarantee the transfer of a particular referral or waiting-list position [26]. Analytically, the quotation exposes the distance between a policy commitment to avoid service-related disadvantage and what the family could use after moving. Promised transfers could fail in concrete ways:

*“They said it would transfer when you move to Portsmouth. It did not.” (P5)*

For families managing multiple co-occurring needs, restarting meant more than repeating paperwork. It involved rebuilding trust with clinicians, re-explaining a child's history and accepting that a wait already endured might not count in the new area. P1 described returning to “the bottom of the waiting list every time we moved” while navigating autism, ADHD, epilepsy and other needs. The interaction between localised service timelines and military mobility made waiting cumulative rather than continuous.

*“We’ve had this huge issue like we need a diagnosis so we can get referral and then we’ve got the referral we can get the help, but then they don’t want to do that because he’s the wrong age and it’s just a whole thing.” (P1)*

Participants’ accounts positioned diagnosis as an institutional passport: it was required to unlock referrals and adjustments, yet age thresholds, local eligibility rules and relocation delayed the production of that evidence. The problem was therefore not delay alone, but a temporal mismatch in which support depended on documentation that the system itself postponed producing. For some families, remaining in one region became a strategy for protecting a fragile clinical pathway, even where doing so constrained service careers.

1.2 Educational Continuity and Transport

Educational mobility had similarly cumulative effects. Parents described repeated school changes, renewed negotiations over Education, Health and Care Plans (EHCPs), and the risk that specialist placements secured through lengthy advocacy would be lost after another posting.

*“He moved to school, I think, in total for 13 times… it really sets the children back each move.” (P11)*

*“And our views as parents did not matter because if we got posted, our children had to go with us, they would not provide us with a retention on the grounds of our child needing consistency of education.” (P5)*

Some families delayed or resisted relocation to preserve educational continuity, while others used tribunals to obtain specialist provision. These accounts suggest that the portability problem extended beyond records: local authority decision-making, school availability and the child's established relationships could not simply be transferred.

School transport further showed how nominal provision could transfer risk and labour back to families. Where children could not reliably communicate distress or safety concerns to unfamiliar drivers, the existence of a taxi did not make the placement accessible.

*“But I actually transport him to and from school on a day basis because I don’t feel that he has a functional communication method to be able to tell the taxi driver.” (P2)*

*“I say the biggest barrier for her is going to get getting in that taxi and getting her there.” (P8)*

P2's decision to drive daily illustrates how a standardised transport offer could externalise safeguarding risk and time costs to the parent. Read alongside P8's account, access depended not only on whether transport existed, but whether it was designed around the child's communication and regulation needs. The consequence was a hidden transfer of labour that constrained paid work and rest, linking educational provision directly to caregiver wellbeing.

2 Parents as Continuity Brokers

When records, referrals and professional understanding did not travel reliably, parents became the connective tissue between otherwise fragmented systems. Advocacy was not described as a single act of speaking up, but as continuing compensatory labour: gathering evidence, learning rules, explaining needs, anticipating disruption, challenging decisions and rebuilding relationships after each move.

2.1 Compensatory Institutional Labour

*“All that fighting we did would have been for nothing because it ended up going back into the tribunal system… And our views as parents did not matter because if we got posted, our children had to go with us.” (P6)*

*“Trust your gut, ask for help… be assertive… advocate constantly for what you and your children need.” (P11)*

The repeated language of “fighting” communicated more than frustration. It described a system in which continuity was treated as an ongoing parental responsibility rather than a property of the service pathway. P11's advice to “advocate constantly” shows how parents came to anticipate that needs would be overlooked without sustained intervention. From a critical-realist perspective, this labour emerged from the interaction between material fragmentation and institutional expectations about parental credibility.

2.2 Contested Recognition, Stigma and Variable Local Discourses

Parents also had to maintain the legitimacy of their child's needs. Private diagnoses could be discounted, while distress or autistic characteristics were sometimes attributed to grief, parenting or behaviour.

*“Certain people don’t accept private diagnosis because they feel like if you’ve paid for it, it doesn’t count.” (P10)*

*“My children were present when we lost my mum. So, they put a lot of it down to grief.” (P3)*

Some accounts described dismissive responses within military workplaces or welfare contexts. P1 recalled a line manager stating that ADHD was “rubbish” and autism did not exist, while P2 felt that their child's needs were treated as a “me problem” rather than an institutional concern. Other participants, however, described commanders or support teams making helpful adjustments. The data therefore do not establish one uniform “tough soldier culture.” They show variable local discourses and practices through which family needs could be legitimised, minimised or made dependent on an individual champion. That variability made support contingent on who interpreted policy at a particular posting.

3 The Unequal Costs of Continuity Work

The work required to secure and maintain support redistributed time, exhaustion, income and career opportunity within families. These consequences were not separate from the support system: they were the costs of compensating for its discontinuities.

3.1 Emotional and Bodily Costs

*“I literally nearly had a breakdown.” (P1)*

*“And for me, I felt I didn’t get a break.” (P10)*

“I was ******* exhausted. I was so tired that I didn’t have the energy.” (P7)

These accounts linked advocacy, daily care and the absence of reliable respite to bodily exhaustion and psychological strain. The costs accumulated across repeated moves, long diagnostic waits and educational disputes. Describing parents as resilient without attending to this transferred labour would risk treating endurance as an individual characteristic rather than evidence of the demands imposed by fragmented systems.

3.2 Occupational and Gendered Costs

Participants connected caregiving and continuity decisions to stalled promotion, career-neutral roles, damaged employment histories and difficulties securing stable housing. One serving parent described remaining in a region to protect a specialist school place and then “not seem[ing] to be going anywhere promotion or career wise” (P7). Another stated:

*“I’ve flown in my career until the point that my daughter’s become ill and now I don’t promote despite doing jobs in higher [roles].” (P12)*

Gendered expectations shaped who was assumed to absorb this work. P1 observed that wives of predominantly male service personnel might give up employment to home educate, whereas, as a serving mother, she did not have a non-serving wife to take on that role. P10 similarly reflected:

*“I think if I was a man, it would be so much easier because if I had a wife, I would settle a wife and children into a permanent home.” (P10)*

The neurodivergence-specific dimension arose from the intersection between military mobility, intensive and often unpredictable care, and gendered assumptions about whose career could be made flexible or secondary.

4 Support as Contingent Relationships

Formal and informal relationships could supply the practical capacity, information and emotional validation that fragmented systems did not. Their value was substantial, but availability depended on proximity, knowledge, willingness and individual discretion. Support was therefore beneficial without being reliably portable.

4.1 Informal Networks as Vital Infrastructure

*“We were quite lucky. We didn’t have so many problems in regard to deployments because we’ve got a really supportive family that would come down and take care of the children.” (P7)*

#### “They… are truly the only way I survive.” (P10)

Relatives, partners, siblings and peers provided respite, school transport, crisis help and a space in which parents could speak without repeatedly justifying their experience. P5 described a sister arriving during difficult days and taking over long enough for the parent to step away; P11 described peer contact as a “safe space to just rant.” These relationships functioned as essential care infrastructure rather than an optional social benefit, particularly during deployment or when formal services were inaccessible.

4.2 Fragility, Uneven Understanding and Institutional Contingency

*“We’ve lost so many friends. I think they just don’t know what to say or they don’t know how to act around it.” (P4)*

*“Some friends have been really understanding… Some of them feel awkward because they don’t really know what to do.” (P1)*

Informal support functioned as both vital infrastructure and a source of vulnerability: families benefited from it, but its availability was voluntary, uneven and geographically fragile. Relocation could remove the relationships that made formal gaps manageable, and limited understanding of neurodivergence could narrow social contact.

Military welfare and community support showed a similar contingency. Some participants felt that staff lacked understanding of hidden disabilities, while others encountered immediate and helpful responses after moving to a different posting. P11 described a base support team becoming involved but added, “If you don't ask, you don't get.” Support could therefore depend on local champions and parental knowledge rather than a consistently accessible institution-wide process.

4.3 Meaning-making, Advocacy Identities and “Little Wins”

Participants also described pride, joy and purpose. Achievements that appeared small within normative developmental expectations could carry particular meaning for families who had invested extensive work in supporting their child.

*“I think with a neurodivergent child, when they achieve something that isn’t necessarily a big deal to a neurotypical child, it’s a bigger win.” (P7)*

*“I just think it’s the little wins.” (P1)*

Some parents transformed accumulated knowledge into advocacy for other families, including signposting to services, helping with EHCP or tribunal paperwork and campaigning for policy change. This meaning-making could be a resource within family stress and resilience processes, but participants resisted uses of resilience that normalised avoidable institutional burden. Their accounts distinguished pride in caregiving and collective advocacy from an expectation that families should simply become more resilient to fragmented provision.

5 Deployment as Redistribution of Care and Regulation

Deployment altered the distribution of care across the family rather than affecting only the absent serving parent. Absence and return changed routines, access to respite, children's regulation and the roles that parents occupied within everyday family life.

5.1 Solo Parenting and Repeated Transitions

*“They get fizzy when he comes back or goes – it takes time to settle. She wouldn’t go to him at all because it was just me.” (P4)*

*“Doing it on your own… you are on your own because a lot of the time in military roles… they can’t have their phone on them.” (P10)*

P4's description of children becoming “fizzy” before absence and after return indicates repeated transition work rather than one bounded period of separation. The parent at home had to maintain routines and respond to anxiety or dysregulation, while the returning parent re-entered a family system that had adapted in their absence. P11 described children moving between anger, reunion and renewed distress during a sequence of departures and returns, showing that the effects were temporal and relational rather than confined to deployment itself.

5.2 Reverberating Wellbeing and Work Impacts

*“What I need is for you to come and look after the kids for a couple hours so I can go and get some sleep because I’ve slept two hours last night.” (P8)*

*“It can have a knock-on effect on you when you’re away with regards to how productive you might be in your job, how you feel from a welfare and mental health perspective.” (P6)*

These accounts show the bodily cost of prolonged solo regulation and the way strain travelled back to the deployed parent through worry, mental health and work productivity. From a family-systems perspective, deployment reorganised routines and roles across the whole family [48–50]. Informal support sometimes buffered these pressures, as in P7's account of relatives providing care during deployments; its absence amplified them. The finding is not that all neurodivergent children respond identically, but that reliance on predictable routines, communication support and familiar attachment relationships made deployment transitions a particularly consequential site of family adjustment.

## Discussion

This study asked what Armed Forces parents and caregivers regarded as usable support, how mobility and deployment shaped its continuity, and how maintaining support affected family life. First, participants described support as effective only when it was accessible, responsive and portable. The nominal presence of a service or policy did not ensure that families could use it. Second, mobility repeatedly interrupted relationships and administrative pathways, while deployment redistributed care and regulation within the family. Formal and informal help could buffer these pressures, but its availability often depended on local knowledge, individual champions and geographic proximity. Third, because systems did not reliably maintain continuity, parents became continuity brokers: they carried information, rebuilt relationships, legitimised needs and coordinated services. This labour had emotional, occupational and financial consequences, even as some parents derived pride, solidarity and purpose from caregiving and advocacy.

### Support as a Contingent Achievement Across Ecological Systems

Consistent with prior work on military families raising autistic children [36–38], relocation was a central barrier to continuity of care. Diagnostic processes, specialist relationships and educational provision were embedded within local organisations and often had to be reconstructed after a move. This aligns with wider evidence of long neurodevelopmental waits and discontinuity across local services [21,39–41], as well as research on the educational effects of military mobility [62–64]. The present analysis extends this literature by identifying support as a contingent achievement rather than a resource that could be inferred from formal eligibility. Parents judged support by whether it remained usable across time and place and whether it reduced, rather than displaced, coordination and risk.

Bronfenbrenner's ecological systems theory helps locate this process across connected levels [47]. Children's daily routines and family relationships were affected by interactions with schools and clinicians; those relationships were shaped by local authorities, NHS trusts, military units and welfare organisations; and these settings operated within wider policy, cultural and geographic conditions. Posting and deployment added a temporal dimension by repeatedly changing the relationships between levels. A critical-realist interpretation further suggests that participants’ experiences were not reducible either to policy texts or to individual perceptions: they arose through encounters between material service arrangements and the meanings through which families and professionals interpreted responsibility [46].

### Parents as continuity brokers and the unequal costs of support

Parents’ extensive advocacy resonates with broader autism-caregiving research in which families learn systems, challenge decisions and repeatedly establish the legitimacy of their child's needs [15,65]. In this study, advocacy had the additional function of maintaining continuity across postings. Parents became repositories of history and coordinators between services that did not reliably communicate. This concept of continuity brokerage links the structural mechanism of fragmentation to the emotional and occupational consequences reported by participants.

Stigma and contested recognition intensified that labour. Participants described parent blame, the discounting of private diagnoses and local responses that treated neurodevelopmental needs as doubtful, temporary or outside military responsibility, consistent with research on professional invalidation and socially shaped understandings of autism [12,66]. The evidence does not justify a general claim that one “tough soldier” culture uniformly governed help-seeking. Rather, it indicates variable local discourses: some personnel dismissed needs, whereas individual commanders and welfare teams sometimes made helpful adjustments. The resulting dependence on local interpretation is analytically important because it made policy implementation uneven.

Occupational effects were also unevenly distributed. Decisions to remain near specialist provision could restrict postings or promotion, while non-serving partners described disrupted employment histories. Participants’ accounts suggest that these costs intersected with gendered expectations about military careers and caregiving [67,68]. The gendered pattern is therefore interpreted specifically through its intersection with neurodivergent caregiving and service mobility.

### Informal and Military Support as Vital but Contingent Infrastructure

Informal networks provided respite, practical help and emotional validation, consistent with evidence that social support can protect caregiver wellbeing and family quality of life [32,69–71]. In military settings, peers and relatives may also understand the demands of mobility and deployment [34,72]. The present findings clarify that informal support was valuable but contingent: its protective capacity depended on proximity, knowledge and willingness, and mobility could remove it abruptly. Families were therefore vulnerable because essential care capacity rested on voluntary and non-portable arrangements rather than because supportive relationships were harmful.

Military welfare showed the same distinction between nominal and usable support. Helpful responses were often attributed to particular commanders, teams or individuals, whereas other participants encountered limited neurodiversity knowledge. This variation supports a shift from relying on exceptional local practice to designing predictable navigation and handover processes. Informal and peer support should complement, not substitute for, accountable formal provision.

### Deployment, Family Systems and a Critical View of Resilience

Deployment-related findings align with family-systems and ecological perspectives that emphasise bidirectional effects between parental absence, child wellbeing and family functioning [50,73,74]. In participants’ accounts, deployment redistributed solo care, sleep loss, emotional regulation and reintegration work. These demands could affect both the parent at home and the serving parent away, including work concentration and mental health. For neurodivergent children who relied on predictable routines or familiar communication partners, transitions around departure and return could be particularly consequential, although experiences varied between families.

Stress and resilience frameworks are useful here when resilience is understood as an interaction among demands, resources, coping and meaning-making rather than as an individual obligation [51–55,75,76]. Participants described pride, “little wins” and advocacy identities that sustained purpose, but some explicitly rejected resilience language when it obscured structural barriers. The finding therefore supports a critical distinction: family adaptation can coexist with harm, and evidence of endurance does not justify underinvestment in portable services or respite [56].

### The policy-implementation gap

The Covenant quotation that support “didn't apply” is best understood as evidence of a policy-implementation gap. The Covenant's due-regard duty asks specified bodies to consider service-related disadvantage in relevant functions, but it does not guarantee a particular referral transfer, waiting-list position or educational placement [26]. Families’ accounts show how broad commitments can fail to become practical continuity when health, education and military systems use separate records, thresholds and local decision processes. The contribution of this study is therefore not that no support exists, but that support remained too dependent on families’ compensatory labour and on local actors’ knowledge.

### Strengths and limitations

Strengths of the study include its focus on an under-researched intersection of military family life and neurodivergence, detailed interviews with serving personnel and spouses or partners, an explicitly reflexive analytic orientation, and careful attention to deductive-disclosure risk in a small and potentially identifiable community. The analysis also distinguishes formal eligibility from support that families experienced as usable and connects system fragmentation to the compensatory work and consequences described across accounts.

The findings are contextually bounded and should not be interpreted as statistically generalisable to all British Armed Forces families. The sample was small and self-selecting and may have attracted caregivers with particularly challenging experiences or established advocacy identities. It did not include all service branches and did not report the full range of rank, ethnicity, socioeconomic position or schooling arrangements. Detailed contextual reporting is therefore intended to support readers’ judgements of transferability rather than population inference. The study reports parental and caregiver accounts and cannot establish children's, practitioners’ or commanders’ perspectives, nor determine the prevalence of the reported experiences or implementation across organisations.

Online interviews improved accessibility for geographically dispersed caregivers but reduced access to embodied and environmental cues compared with in-person fieldwork. The extended recruitment and fieldwork period may also have encompassed changes in local service conditions. Rank and children's schooling arrangements were not considered in the analysis due to the unacceptable risk of participant identification. However, these factors may have significantly influenced parents’ perceptions and experiences. Finally, the interview guide did not systematically ask participants to articulate a philosophical position on neurodiversity. The analysis should therefore not be read as attributing one shared position to all families.

Future research should include neurodivergent children and young people, practitioners, welfare personnel and commanders; compare experiences across service branches, ranks and posting patterns; examine intersectional differences in access and caregiving; and prospectively evaluate specific continuity, navigation and respite interventions. Such work should prioritise analytic transferability and mechanism rather than treating a larger qualitative sample as a route to statistical generalisation.

### Implications for policy and practice

The findings support a shift from nominal eligibility to continuity-by-design. Recommendations are directed at the mechanisms identified in participants’ accounts; the qualitative study does not establish the effectiveness or cost-effectiveness of any particular intervention, and the proposals require prospective evaluation.

### Continuity across health and education systems

Health and education bodies should establish explicit inter-area handover procedures that preserve referral evidence, assessment history and current provision when families move. Nationally portable records alone will not resolve local differences, but accountable transfer protocols could reduce repeated evidence-gathering and make responsibility for follow-up visible. Schools and local authorities should plan SEND transitions before a posting where possible and distinguish support funded through the Service Pupil Premium from statutory SEND duties [27].

### Navigation and workforce knowledge

Defence and welfare organisations should provide a named navigation contact before and after posting, with neurodiversity-informed training and sufficient authority to coordinate across local health, education and welfare systems. Training for military welfare staff, school special educational needs coordinators and healthcare professionals should be co-developed with neurodivergent people and families and should address hidden disability, communication differences, diagnostic legitimacy and the practical effects of mobility [77,78].

### Deployment-sensitive support

Units and welfare providers should anticipate periods of elevated solo-care burden before, during and after deployment. Where feasible, flexible work, reliable signposting, planned check-ins and access to appropriate respite or childcare could reduce sleep loss and protect caregiver and serving-person wellbeing. Existing evidence suggests that respite may be associated with lower stress, anxiety and depression among military parents raising autistic children, although further evaluation is required [79].

### Informal networks and peer support

Peer groups, online networks and family education can provide validation, practical knowledge and continuity across postings. These resources should be supported as complements to formal provision, not used to transfer statutory or organisational responsibility to volunteers and relatives. Mobility-aware initiatives should consider how families can retain contact with trusted networks while also building relationships at a new posting.

## Conclusion

For the families in this study, support was not experienced as a stable resource that simply existed within formal or informal networks. It had to be made continuous across systems designed around stable residence and ordinary family availability. Mobility disrupted diagnostic, educational and clinical pathways; parents compensated by becoming continuity brokers, often at substantial emotional, occupational and financial cost. Military welfare, commanders, relatives and peers could provide decisive help, but support remained contingent on knowledge, relationships and proximity. Deployment further redistributed care and disrupted family regulation. A neurodiversity-informed response therefore requires more than encouraging parental resilience: it requires portable records and decisions, accountable handovers, knowledgeable welfare provision and support that anticipates the temporal demands of posting and deployment.

## Supporting information

S3 File Interview Schedule. The interview schedule has been uploaded as a separate Supporting Information file.
(DOCX)

S1 Table De-identified example of the route from participant extract to initial code, analytic interpretation and final theme. (DOCX)

S2 File Standards for Reporting Qualitative Research (SRQR) checklist. The completed 21-item SRQR checklist has been uploaded as a separate supporting information file.
(DOCX)
