# Supplementary material for: Navigating fragmented and contingent support: A qualitative study of British Armed Forces families raising neurodivergent children
Source: PLoS One. 2026 Sep 21;21(9):e0358595. doi: 10.1371/journal.pone.0358595 (PMC13592621; doi:10.1371/journal.pone.0358595)
Supplement: Supplementary file 1 [file pone.0358595.s001.docx]

**Interview Schedule**

**1. Diagnosis**

**Main question:**

- Can you tell me about any experiences your family has had with recognising neurodivergence and with assessment, diagnosis, or formal identification?

**Possible prompt questions:**

- How did this process begin?
- What happened during the process?
- What, if anything, was helpful?
- What, if anything, was difficult?
- Was an assessment or diagnosis sought? What factors influenced that?
- How did the experience affect you and your family?
- Has military life influenced the process in any way?

**2. Military family and military community**

**Main question:**

- Can you tell me what being part of a military family has been like for you and your family?

**Possible prompt questions:**

- What aspects of military family life have been most significant for your family?
- How, if at all, has the military community influenced your family’s experience of neurodivergence?
- Can you describe any particularly supportive or difficult experiences?
- How understood or included has your family felt within the military community?
- What role have other military families played in your family’s life?

**3. Relocation**

**Main question:**

- Can you tell me about your family’s experiences of military-related moves or relocations?

**Possible prompt questions:**

- What impact, if any, have relocations had on your family?
- What has been difficult about relocating?
- What, if anything, has been positive?
- How have relocations affected routines, relationships, or family wellbeing?
- How have relocations affected access to assessment, healthcare, education, or other support?
- What has helped your family manage transitions?

**4. Children's Education**

**Main question:**

- How, if at all, has military life affected your child or children’s education and educational support?

**Possible prompt questions:**

- Can you tell me about their experiences of changing schools or educational settings?
- How have relocations affected continuity in their education?
- How have schools responded to their needs?
- What has helped maintain continuity?
- What has made continuity difficult?
- Have educational records, assessments, or support arrangements transferred successfully?
- What support would have made school transitions easier?

**5. Employment and Career Prospects**

**Main question(s):**

- How, if at all, has military life affected your own employment, education, or career plans?
- How, if at all, has military life affected your partner or spouse’s employment or career, where applicable?

**Possible prompt questions:**

- Can you describe any opportunities you have experienced?
- Can you describe any difficulties?
- Have relocation or caring responsibilities affected employment decisions?
- Have your family’s neurodiversity-related needs affected your employment or career?
- What employment-related support, if any, has been available?

**6. Formal and Informal Support**

**Main question – Formal support:**

- What formal services or professional support, if any, has your family used or currently uses in relation to neurodivergence? such as the types of professionals (e.g., social workers, therapists etc.) or organisations (e.g., charities, NHS mental health services).

**Possible prompt questions:**

- How did you find out about it?
- How easy or difficult was it to access?
- How helpful or appropriate was it?
- How well was support maintained during relocations?
- How well did military and civilian services work together?

**Main question – Informal support:**

- What informal support, if any, has your family received from relatives, friends, peers, or other military families?

**Possible prompt questions:**

- What kind of support was provided?
- How helpful was it?
- Was any informal support unhelpful or difficult?
- Has relocation affected your informal support network?
- Have you been able to connect with other families with similar experiences?

**7. Accessibility of support**

**Main question**

- What, if anything, has made it easier or harder for your family to find, access, and continue receiving appropriate support?

**Possible prompt questions:**

- Awareness of available services
- Referral procedures
- Eligibility criteria
- Waiting times
- Location and travel
- Relocations or postings
- Coordination between military and civilian services
- Professionals’ understanding of military family life
- Professionals’ understanding of neurodivergence

**8. Gaps and additional support**

**Main question(s)**

- What support, if any, do you feel is currently missing, insufficient, or in need of improvement?
- What would appropriate and useful support look like for your family?

**Possible prompt questions:**

- What type of support would be useful?
- When should that support be offered?
- Who should provide it?
- How should it be delivered?
- How should support be maintained during relocations?
- What should military organisations or civilian services do differently?
