# Supplementary material for: Navigating fragmented and contingent support: A qualitative study of British Armed Forces families raising neurodivergent children
Source: PLoS One. 2026 Sep 21;21(9):e0358595. doi: 10.1371/journal.pone.0358595 (PMC13592621; doi:10.1371/journal.pone.0358595)
Supplement: Supplementary file 2 [file pone.0358595.s002.docx]

S1 Table. De-identified example of the route from participant extract to initial code, analytic interpretation and final theme.

| **Participant extract** | **Initial code and analytic memo** | **Candidate pattern** | **Final theme** |
| --- | --- | --- | --- |
| P2: daily school transport was rejected because the child lacked a functional way to tell the driver about distress or risk. | Generic transport perceived as unsafe; nominal provision transfers safeguarding responsibility and time to the parent. | Provision exists but is unusable without communication-sensitive design. | 1. Support made non-portable by mobility |
| P1: a diagnosis was needed for referral, but age thresholds delayed the diagnosis. | Diagnosis operates as institutional currency; support depends on evidence the system postpones producing. | Temporal gatekeeping and cumulative waiting. | 1. Support made non-portable by mobility |
| P11: parents must "advocate constantly". | Continuity is treated as parental responsibility; advocacy replaces missing coordination between systems. | Compensatory institutional labour. | 2. Parents as continuity brokers |
| P10: close others were "the only way I survive". | Informal help supplies essential care capacity, but depends on proximity and willingness. | Vital but non-portable relational support. | 4. Support as contingent relationships |
| P4: children became "fizzy" when the serving parent left or returned. | Departure and reintegration require repeated regulation and role adjustment across the family. | Deployment reorganises routines and care. | 5. Deployment as redistribution of care and regulation |
