# Supplementary material for: Navigating fragmented and contingent support: A qualitative study of British Armed Forces families raising neurodivergent children
Source: PLoS One. 2026 Sep 21;21(9):e0358595. doi: 10.1371/journal.pone.0358595 (PMC13592621; doi:10.1371/journal.pone.0358595)
Supplement: Supplementary file 3 [file pone.0358595.s003.docx]

**S2 Checklist. SRQR checklist**

*Navigating fragmented and contingent support: A qualitative study of British Armed Forces families raising neurodivergent children*

**Reporting guideline:** Standards for Reporting Qualitative Research (SRQR)

| **SRQR item** | **Reporting Standard** | **Location in Manuscript**  **(Section/Page)** | **Evidence from Manuscript** | **Compliance**  **Status** |
| --- | --- | --- | --- | --- |
| S1. Title | The title should concisely identify the study topic and indicate that the study is qualitative or name the qualitative approach or data-collection method. | Title page, p. 1, lines 2-3; article title, p. 2, lines 24-25. | The title identifies the population and phenomenon and explicitly labels the work as "A qualitative study" of British Armed Forces families raising neurodivergent children. | Complete |
| S2. Abstract | The abstract should summarise the problem or background, purpose,  methods, principal findings, and conclusions or implications. | Abstract, p. 2, lines 26-47. | The abstract reports the military-family context, study purpose, 12 participants, online semi-structured interviews, critical-realist orientation, reflexive thematic analysis, principal findings, and policy and practice implications. | Complete |
| S3. Problem formulation | The report should define the problem, establish its significance, review  relevant empirical and theoretical work, and identify the knowledge gap. | Introduction, pp. 3-7, lines 50-154. | The Introduction defines neurodivergence and usable support, reviews caregiver burden, mobility, deployment, formal and informal support, UK military policy, and relevant empirical and theoretical work. It identifies a process-level gap concerning support usability, continuity across local systems, and who absorbs coordination work. | Complete |
| S4. Purpose or research question | The study purpose and specific objectives or research questions should be stated clearly. | The Present Study, p. 7, lines 155-164. | The manuscript states the study aim and three research questions addressing adequate and usable support, the effects of mobility and deployment on continuity, and consequences for wellbeing, resilience, occupational trajectories, and family life. | Complete |
| S5. Qualitative approach and research paradigm | The qualitative methodology, research  paradigm, guiding theory, and the rationale for their use should be identified. | Introduction, pp. 6-7, lines 142-154;  Methods - Design and Epistemology, pp. 7-8, lines 165-174. | The study is identified as qualitative and uses Braun & Clarke's reflexive thematic analysis within a critical-realist orientation. Ecological systems, family-systems, and stress-resilience concepts are identified as later sensitising lenses rather than a predetermined deductive codebook. | Complete |
| S6. Researcher characteristics and reflexivity | Report researcher characteristics, experience, assumptions,  relationships with participants, and how these may have shaped the questions, methods, analysis, and transferability. | Researcher Positioning and Reflexivity, pp.  8-9, lines 175-198; Data Analysis and  Analytic Quality, p. 13, lines 289-314. | The manuscript reports that three interviewers came from British military families and had qualitative and neurodiversity research experience; it acknowledges that familiarity could shape questioning and interpretation. Reflective notes, reflexive memos, team  discussions, and audit notes are described, and NK is identified as lead analyst. | Complete |
| S7. Context | Describe the setting and salient social, organisational, geographical, temporal, or policy factors that shaped  the study, with a rationale where relevant. | Introduction, pp. 3-7, lines 50-154;  Participants and Recruitment, pp. 9-10, lines 197-230; Data Collection, p. 11, lines 241-252. | The report situates the study within the UK Armed Forces context and explains mobility, deployment, fragmented NHS and local-authority systems, schooling, military welfare, and relevant policy arrangements. It also explains the use of remote  interviews for geographically dispersed families with service and caregiving responsibilities. | Complete |
| S8. Sampling strategy | Explain how and why participants or other units were selected, the stopping or adequacy criterion, and the rationale for the sampling approach. | Participants and Recruitment, pp. 9-10, lines 197-230; Sample Adequacy, p. 10,  lines 231-240. | Recruitment was convenience- and volunteer-based through an online survey, military networks, support-organisation social media, and participant referral. All 12 eligible volunteers were interviewed, no purposive selection was applied, and adequacy was justified using information power. | Complete |
| S9. Ethical issues pertaining to human subjects | Report ethics approval, consent, confidentiality, data security, and any  additional protections relevant to participants. | Ethical Considerations, p. 12, lines 273-  288; Data Availability Statement, pp. 29-30, lines 704-715. | The manuscript provides the University of Lincoln ethics approval number, written consent procedures, recording and quotation consent, withdrawal rights, distress-support information, pseudonymisation, deductive-disclosure safeguards, quotation review, controlled-access arrangements, secure institutional storage, and a retention period. | Complete |
| S10. Data collection methods | Describe the types of data collected, collection procedures, timing,  iterative or adaptive processes, any triangulation, and the rationale for the chosen method. | Participants and Recruitment, p. 10, lines 216-230; Data Collection, p. 11, lines 241-264. | The report describes the broad data-collection period, online semi-structured interviews through Microsoft Teams, 60-90-minute duration, video or audio-only  choice, field notes, core topic areas, a flexible interview format, and the accessibility rationale. | Complete |
| S11. Data collection instruments and technologies | Describe the interview guide or other instruments, recording technologies, and whether instruments changed during the study. | Data Collection, p. 11, lines 241-264;  Transcription and Data Preparation, p. 12, lines 265-272. | The interview guide was co-developed by the research team and informed by a systematic review and prior survey findings; its main topic areas are listed. Microsoft Teams, optional video, audio recording, field notes, and NVivo are identified. A full interview guide is listed in the Supporting Information. | Complete |
| S12. Units of study | Report the number and relevant characteristics of participants or other units and the level of participation. | Participants and Recruitment, pp. 9-10, lines 198-230; Table 1, p. 9; Strengths and  Limitations, p. 27, lines 636-640. | The manuscript reports 12 participants, gender distribution, age range, relationship to service, service contexts, child age range, neurodevelopmental and co-occurring needs, and completion of all 12 interviews. Rank, schooling arrangements, ethnicity, and socioeconomic position are withheld in Table 1 for deductive-disclosure reasons. | Complete |
| S13. Data processing | Describe transcription, data preparation, management and security, integrity checks, coding preparation, and anonymisation or de-  identification. | Transcription and Data Preparation, p. 12, lines 265-272; Ethical Considerations, p.  12, lines 273-288; Data Availability  Statement, pp. 29-30, lines 704-715. | Initial transcripts were generated from Microsoft Teams recordings, prepared as verbatim records, checked against the audio by NK and MD, corrected, de-identified, and managed in NVivo. Locations, ranks, units, and rare combinations were removed or generalised; quotation-level disclosure review and secure institutional storage are reported. | Complete |
| S14. Data analysis | Explain how codes, categories, interpretations or themes were generated and refined, who participated in analysis, and how the  approach related to the paradigm. | Data Analysis and Analytic Quality, p. 13, lines 289-314; S1 Table, p. 30, lines 716-719. | The manuscript reports the six phases of reflexive thematic analysis, line-by-line coding of meaning-bearing units, movement from primarily inductive coding to latent patterned meanings, cross-case comparison, clustering into candidate patterns,  central organising concepts, whole-dataset theme review, NK as lead analyst, and reflexive challenge by the wider team. S1 Table illustrates the route from extract to code, candidate pattern, and final theme. | Complete |
| S15. Techniques to enhance trustworthiness | Describe and justify procedures used  to strengthen credibility, transparency,  dependability, or reflexive  accountability. | Researcher Positioning and Reflexivity, pp.  8-9, lines 187-198; Data Analysis and  Analytic Quality, p. 13, lines 300-314. | Reported procedures include reflective notes, reflexive memoing, team discussions, audit notes, repeated engagement with the complete dataset, checking all five themes against all 12 transcripts, attention to recurrent and less common accounts, and a  participant-by-quotation audit. These procedures support transparency and reflexive accountability rather than coder-reliability testing. | Complete |
| S16. Synthesis and interpretation | Present the principal findings as a coherent synthesis of interpretations, themes, inferences, concepts, or  models. | Results, pp. 14-22, lines 317-524;  Discussion, pp. 22-26, lines 525-620. | Five interlocking themes are presented with central organising concepts and analytic interpretation: non-portable support, parents as continuity brokers, unequal costs, contingent relationships, and deployment as redistribution of care and regulation. The Discussion synthesises the findings through the research questions and theoretical lenses. | Complete |
| S17. Links to empirical data | Provide empirical material, such as  quotations or excerpts, that substantiates the analytic claims. | Results, pp. 14-22, lines 317-524;  participant identifiers P1-P12; S1 Table, p. 30, lines 716-719. | Each theme is supported by attributed participant quotations followed by analytic commentary. Participant identifiers preserve traceability without disclosing identity. S1 Table links selected extracts to initial codes and analytic memos, candidate patterns,  and final themes. | Complete |
| S18. Integration with prior work, implications, transferability, and contribution | Summarise the findings, relate them to prior scholarship and theory, explain implications and scope of transferability, and identify the study  contribution. | Discussion, pp. 22-26, lines 525-620; Strengths and Limitations, pp. 26-27, lines  621-650; Implications for Policy and  Practice, pp. 27-28, lines 651-684;  Conclusion, p. 29, lines 685-696. | The Discussion returns to the research questions, relates findings to military-family, autism-caregiving, social-support, stigma, gender, deployment, and resilience scholarship, and applies critical realism and ecological and family-systems concepts. It identifies continuity brokerage and contingent support as contributions, bounds transferability, and develops policy and practice implications. | Complete |
| S19. Limitations | Discuss limitations affecting the trustworthiness, scope, interpretation, and transferability of the findings. | Strengths and Limitations, pp. 26-27, lines 621-650. | The manuscript addresses the small self-selecting sample, possible over-representation of challenging experiences or advocacy identities, incomplete service-  branch and demographic coverage, reliance on caregiver accounts, inability to estimate prevalence or organisational implementation, limitations of online interviews, the extended fieldwork period, and the absence of systematic questioning about philosophical positions on neurodiversity. | Complete |
| S20. Conflicts of interest | Disclose potential or perceived conflicts of interest and explain how they were managed. | Completed in PLOS ONE submission system | Handled in the submission metadata, not required in manuscript text. | Complete (system) |
| S21. Funding | Report funding and material support and describe the funder's role in the study and publication process. | Completed in PLOS ONE submission system | Handled in the submission metadata. No Funding. | Complete (system) |
